# Digital Dating Abuse: An Application of the Theory of Planned Behavior

**DOI:** 10.1177/08862605231205595

**Published:** 2023-10-21

**Authors:** Jennifer McArthur, Julie Blais, Marguerite Ternes

**Affiliations:** 1 Dalhousie University, Halifax, NS, Canada; 2 Saint Mary’s University, Halifax, NS, Canada

**Keywords:** Digital dating abuse, dating violence, emerging adults, theory of planned behavior

## Abstract

Social media and other technologies are being increasingly adopted as mechanisms to perpetrate abuse against dating partners. Using Ajzen’s theory of planned behavior as a framework, a sample of 352 emerging adults completed a questionnaire that assessed the core constructs of the theory of planned behavior (i.e., attitudes, subjective norms, perceived behavioral control), as well as intentions to commit three types of digital dating abuse in the near future: digital monitoring and control, digital direct aggression, and digital sexual coercion. The models explained 44%, 34%, and 44% of the variance in intentions to commit digitally facilitated monitoring and control, direct aggression, and sexual coercion, respectively. Attitudes and subjective norms significantly predicted intentions, whereas perceived behavioral control did not. Given the increasing prevalence of digital dating abuse, prevention efforts should target attitudes toward digital dating abuse-related behaviors and perceptions of social acceptability and engagement.

## Introduction

In 2018, Inyoung You was sending her boyfriend, Alexander Urtula, almost 800 text messages a day (Taylor, 2019). It was a constant and inescapable barrage of cruel messages—You repeatedly called her boyfriend “worthless,” threatened physical violence, and urged him to “do everyone a favor and go [*expletive*] kill [him]self.” After 2 months and more than 47,000 text messages from You, Urtula took his own life on the day he was set to graduate from Boston College. Although extreme, the You/Urtula case highlights the emerging trend of digital dating abuse (DDA), which encompasses three broad types of partner aggression perpetrated using technologies: digital monitoring or control (e.g., excessive text messaging), digital direct aggression (e.g., direct threats of violence), and digital sexual coercion (e.g., pressuring a partner to send a sext; Reed et al., 2016). Estimates of DDA vary considerably, however, upwards of 93% of undergraduate students report experiencing or engaging in at least one instance of online aggression in their current relationship (Leisring & Giumetti, 2014), suggesting that these behaviors have become increasingly common.

Despite the persistent belief that cyber-based violence is harmless, at least compared to physical violence (Stonard et al., 2017), empirical studies have documented a myriad of adverse psychological effects associated with cybervictimization, such as low self-esteem, depression, suicide ideation, and maladaptive coping behaviors (e.g., substance use; Gracia-Leiva et al., 2020; Marganski & Melander, 2018). High prevalence rates combined with these negative outcomes signal a growing need to identify and evaluate factors associated with emerging adults’ perpetration of DDA which may be targeted through prevention efforts. Ajzen’s theory of planned behavior (TPB) has been well validated for studying a number of different antisocial behaviors (e.g., dating violence; Betts et al., 2011; Hou et al., 2020; Tolman et al., 1996) and may therefore be a useful framework for identifying factors related to DDA perpetration. To this end, the current study applied the TPB to assess the degree to which attitudes, subjective norms, and perceived behavioral control were associated with emerging adults’ intentions to perpetrate digital monitoring or control, digital direct aggression, and digital sexual coercion against a current or former partner, with the ultimate goal of identifying potentially malleable factors to aid in the development of prevention and intervention efforts.

### Digital Dating Abuse

The three types of DDA—digital monitoring or control, digital direct aggression, and digital sexual coercion—vary in terms of behaviors, prevalence, and motivations. Often considered the most pervasive form of DDA (e.g., Borrajo et al., 2015; Gracia-Leiva et al., 2020), *digital monitoring or control* is characterized by overt or covert intrusive behaviors aimed at controlling or intimidating a dating partner, such as repeatedly calling or texting a partner or accessing accounts without permission (Reed et al., 2016). Although evidence suggests that these behaviors can be motivated by jealousy, recent studies have also noted a social shift where certain monitoring behaviors, such as excessive texting, are perceived as normative in relationships, rather than intrusive (Reed et al., 2016; Stonard et al., 2017). *Digital direct aggression* is often motivated by anger (Reed et al., 2021) and refers to the use of social media and communication technologies to harass a dating partner privately or publicly and can include minor aggressions, such as swearing or insulting a partner through text or email, to more severe forms, including sending threats or using social media to publicly humiliate a partner (Leisring & Giumetti, 2014; Reed et al., 2016). Roughly one in ten American and Canadian undergraduate students report being a victim of such abuse (e.g., Hancock et al., 2017; Leisring & Giumetti, 2014; Zapor et al., 2017). Finally, *digital sexual coercion* is characterized by unwanted sexual behavior conducted by electronic means and may include pressuring a partner to send a sexually explicit message or nude image/video (i.e., coerced sexting) or disseminating sexually explicit images or videos of a partner without permission (Powell & Henry, 2019; Reed et al., 2016). Although seemingly less prevalent than digital-monitoring behaviors and digital aggression, an estimated 20% of young adults have felt pressured into sexting by a dating partner (Drouin et al., 2015) and over 10% of college women and 6% of college men have reported being pressured by a partner to engage in sexual activities via technology (Reed et al., 2016).

Previous research has examined different types of correlates associated with DDA perpetration. Studies exploring the effects of gender on DDA perpetration have produced mixed findings. Some studies suggest that young adult men and women perpetrate DDA at similar rates (Burke et al., 2011; Leisring & Giumetti, 2014); however, other studies indicate gender disparities across the various behaviors, with women more likely to perpetrate digital monitoring and control (Borrajo et al., 2015) and men more likely to perpetrate sexually coercive behaviors in digital environments (Reed et al., 2016). Studies examining behavioral correlates indicate a relationship between alcohol use and DDA perpetration, although this may vary as a function of gender and disposition. In a sample of 258 U.S. college students, Brem et al. (2021) found that alcohol use positively predicted DDA perpetration in women, but not men, with high traits of romantic jealousy. Other studies have demonstrated that personality factors, such as psychopathy (e.g., callousness, impulsivity) and vulnerable narcissism (e.g., hypersensitivity; fragile ego), are also associated with increased perpetration of DDA (Branson & March, 2021).

Despite the increased interest in identifying correlates of DDA, few studies have taken a theoretical approach to better understand why certain factors may influence emerging adults’ decision to engage in DDA. Utilizing a well-validated theoretical framework would increase the validity of the findings and may also increase consistency between studies. The TPB is a well-established behavioral theory that has been previously used to explain the perpetration of antisocial behaviors, such as offline dating violence (e.g., Betts et al., 2011; Kernsmith, 2005; Kernsmith & Tolman, 2011; Tolman et al., 1996) and cyberbullying (Heirman & Walrave, 2012; Pabian et al., 2014) and may therefore provide a useful organization framework for explaining intentions to perpetrate DDA.

### Theory of Planned Behavior

According to the TPB, behavioral intentions and, in turn, the performance of any behavior are motivated by three factors: attitudes toward the behavior (i.e., the favorable or unfavorable evaluation of performing the behavior), subjective norms (i.e., the perception of social pressure to perform a behavior), and perceived behavioral control (i.e., the perception that one can perform the behavior; Ajzen, 1991). Behavioral intentions have been shown to reliably predict actual behaviors with an average correlation of .47 (*R*^2^ = .22) between the two variables (Armitage & Conner, 2001). While the TPB has shown utility in explaining dating violence in offline contexts (Betts et al., 2011; Hou et al. 2020) and cyberbullying (Heirman & Walrave, 2012), few studies have applied it to DDA. One notable exception is Darvell et al. (2011) who examined the influence of attitudes, subjective norms, and perceived behavioral control on partner-monitoring behaviors among 244 university students. Results indicated that more accepting attitudes toward monitoring behaviors, along with increased social pressure from friends, were associated with intentions to monitor a partner’s activity on Facebook. To our knowledge, no studies have applied the TPB to digital-monitoring and control behaviors beyond Facebook, nor has the TPB been applied to digital direct aggression and digital sexual coercion in the context of young adults’ intimate relationships. Several studies, however, have examined the association between some of the individual components of TPB and DDA, despite not utilizing the full model.

#### Attitudes Toward DDA

Attitudes are an individual’s favorable or unfavorable evaluation of behavior, which are guided by the potential positive or negative outcome associated with performing the behavior (Ajzen, 1991). Attitudes have emerged as one of the most consistent predictors of partner violence (Betts et al., 2011; Hou et al., 2020), and increasing evidence suggests that more favorable attitudes are also associated with the perpetration of DDA. For example, young adults who equated digital monitoring and control with love and affection engaged in higher levels of behavior than young adults who did not hold the same attitudes (Borrajo et al., 2015). Attitudes have also been associated with the non-consensual dissemination of another’s intimate image, a behavior most likely to be committed against a former partner. Clancy et al. (2020), for instance, found that university students were more likely to have previously engaged in non-consensual dissemination when they viewed the act as humorous. Overall, more positive attitudes are related to increased engagement in various DDA behaviors.

#### Perceived Norms

In addition to one’s attitudes, social pressures (i.e., subjective norms in the TPB model) and one’s social environment appear to play an important role in the decision to perpetrate violence. A meta-analysis by Park and Kim (2018), for instance, identified 131 family- and community-related risk factors across 27 studies; family relationship problems, witnessing partner violence, and deviant peers emerged as the strongest predictors of committing intimate partner violence. Although limited, preliminary findings suggest that perceived norms are also a key predictor of DDA perpetration. Like Darvell et al. (2011), who found that subjective norms were the strongest TPB predictor of partner monitoring, Van Ouytsel et al. (2020) found perceived social norms (i.e., the belief that peers were engaging in similar behaviors) to be the strongest predictor of young adults’ engagement in digital-monitoring behaviors in their application of social learning theory. Likewise, subjective norms have emerged as an important explanatory variable associated with sexting behavior (Hudson et al., 2014) and the non-consensual dissemination of intimate images (Clancy et al., 2019) among undergraduate students.

#### Perceived Behavioral Control

According to the TPB, behavioral intentions and decisions are also guided by perceived behavioral control, or an individual’s perception that the performance of a behavior will be easy or difficult (Ajzen, 1991). While there is evidence of a significant relationship between perceived behavioral control and dating violence (Betts et al., 2011; Hou et al., 2020), its role in DDA remains unclear. In the sole study examining the TPB’s influence on DDA, perceived behavioral control failed to explain partner monitoring on Facebook (Darvell et al., 2011). Similarly, TPB-informed research on cyberbullying suggests a lack of association between perceived behavioral control and online aggression (Heirman & Walrave, 2012; Pabian & Vandebosch, 2014), leading some researchers to conclude that perceived behavioral control does not matter when nearly all youth and young adults possess the ability to engage in cyberaggression given the ubiquity of technology usage (Doane et al., 2014).

It is also possible that the digital environment itself may facilitate the perpetration of DDA, thereby increasing perceptions of behavioral control. Communication-related aspects unique to technology, such as perceived anonymity, asynchronous communication, and a perceived lack of authority, may lead to increased behavioral disinhibition in online environments (Suler, 2004). For instance, online communication typically occurs asynchronously via text, prompting less reservations about engaging in hurtful behaviors as the victim’s emotional response is concealed behind a screen. The use of technology may therefore make it easier for some individuals to avert responsibility and engage in aggressive behavior that they would not otherwise commit when face-to-face with a partner. However, given its significant association with partner violence (Betts et al., 2011), further exploration of the effect of perceived behavioral control on DDA behaviors such as digital direct aggression and digital sexual coercion is warranted.

### Current Study

The goal of the current study was to evaluate the degree to which attitudes, subjective norms, and perceived behavioral control influenced intentions to perpetrate three types of DDA: digital monitoring and control, digital direct aggression, and digital sexual coercion. Based on theoretical (e.g., Ajzen, 1991) and empirical (e.g., Betts et al., 2011; Darvell et al., 2011) evidence, attitudes toward DDA, subjective norms, and perceived behavioral control, independently, and in combination, were expected to positively predict emerging adults’ behavioral intentions to perpetrate all three types of DDA. Given the significant implications of DDA, it is important to identify factors that are significantly related to DDA perpetration and that could potentially be used in the development of effective prevention and intervention programs. Utilizing the well-established theoretical framework of the TPB also has the added benefit of situating our potential findings within the boarder literature on behavioral change, thereby allowing us to make clearer recommendations for preventing future DDA perpetration.

## Method

### Participants and Procedure

Emerging adults between the ages of 18 and 25 who had been in at least one romantic relationship since the age of 18 were recruited from two Atlantic Canadian university participant pools (i.e., SONA) from September 2021 to April 2022. The final sample (*N* = 352) included 301 women and 51 men with a mean age of 20.13 (*SD* = 1.75). An a priori power analysis was conducted using G*Power (version 3.1; Faul et al., 2007) to determine the required sample size for multiple linear regression with four predictor variables, a power of .80, and an alpha value of .05. The power analysis estimated a sample of 85 participants to achieve a medium (*f*^2^ = 0.15) effect. Most participants identified as non-Hispanic White (77.8%), followed by Asian (6.3%) and Black (5.4%). Over two-thirds (71.0%) of participants identified as heterosexual and 20.0% identified as bisexual. In terms of current relationship status, most participants reported currently being in a dating relationship (55.1%), followed by single (37.8%), married (5.4%), and other (1.7%; e.g., casually dating). A comparison of intentions between participants in a relationship and participants who were single at the time of the study is provided in Supplemental S1 (p. 1).

After the informed consent, participants were provided with a definition of a dating partner at the start of the survey. In the current study, dating partner could refer to: “. . . ANY of the following: a boyfriend or girlfriend, someone you are a ‘thing’ with, someone you have dated or are currently dating (e.g., going out without being supervised), someone who you like or love and spend time with, or a relationship that might involve sex” (Reed et al., 2016). Participants were then asked whether, based on the definition, they have had at least one dating partner since the age of 18. As DDA can be committed against a current or former partner, participants who responded with “yes” completed an online study hosted via Qualtrics and received 0.5 bonus points toward an eligible psychology course. Those who responded with “no” were screened out of the study. The study was approved by both university ethics boards (REB# 21-109 and REB #2021-5821).

### Measures

Following recommendations from Ajzen (2006), an author-defined scale was developed to assess the central TPB constructs—attitudes toward DDA, subjective norms, perceived behavioral control, and behavioral intentions to commit the DDA in the near future. Participants rated the extent to which each TPB construct applied to the different types of DDA perpetration. In total, 18 behaviors representing each type of DDA were included based on Reed et al.’s (2016) digital dating abuse perpetration subscale: digital monitoring and control subscale (six items; e.g., pressuring a partner to respond quickly to texts and calls; monitoring who the partner is friends with on social media), digital direct aggression (eight items; e.g., posting mean or hurtful messages about partner to social media; using a cell phone or internet to send partner threatening messages), and digital sexual coercion (four items; e.g., pressure partner to sext; sent intimate image of partner to others without partner’s consent). Participants were not instructed to think about any specific partner when responding to the remaining questions in the survey. See section “Method” of the Supplemental Materials for all DDA behaviors and items.

#### Behavioral Intention to Perpetrate

Behavioral intentions were assessed by evaluating a participant’s expectation of perpetrating each of the 18 DDA behaviors using the internet or cell phone in the next 4 weeks on a 7-point scale from 1 (*extremely unlikely*) to 7 (*extremely likely*). Example items included the following: “In the next 4 weeks, I will monitor my partner’s whereabouts and activities; pressure my partner to sext.” Scores for intentions were computed by averaging responses across the three forms of DDA with higher scores representing higher intentions to perpetrate digital monitoring and control, digital direct aggression, and digital sexual coercion, respectively. Internal consistency for intentions was high when assessed by DDA type: digital monitoring and control (α = .81); digital direct aggression (α = .77); and digital sexual abuse (α = .77).

#### Attitudes Toward DDA

Attitudes were measured by assessing the degree to which participants favorably evaluated the perpetration of each of the 18 DDA behaviors. Participants responded to two 7-point semantic differential scales designed to measure affective and instrumental aspects of attitudes (e.g., “To me, pressuring my partner to respond quickly to calls, texts, or other messages is. . .” *unenjoyable* (1) to *enjoyable* (7); and *harmful to me* (1) to *beneficial to me* (7). For each behavior, the two attitude items were averaged, and composite scores were computed for the three forms of DDA with higher scores indicating more favorable attitudes toward the perpetration of digital direct aggression, digital monitoring and control, and digital sexual coercion, respectively. High internal consistency for attitudes was demonstrated when assessed by DDA type: digital monitoring and control (α = .88); digital direct aggression (α = .86); and digital sexual coercion (α = .79).

#### Subjective Norms

Subjective norms were measured by evaluating perceptions of others’ approval to engage in DDA behaviors (i.e., injunctive norms) and perceptions of others’ behavior (i.e., descriptive norms). Each of the 18 DDA behaviors consisted of two parallel items: one for injunctive norms (e.g., people who are important to me think I should monitor who my partner talks to and is friends with) and one for descriptive norms (e.g., people who are important to me monitor who their partners talk to and is friends with). All items were measured on a seven-point Likert scale ranging from 1 (*strongly disagree*) to 7 (*strongly agree*). Injunctive and descriptive norms were averaged for each behavior and composite subjective norms scores were computed for the three forms of DDA by averaging across all the behaviors for each of the three types of DDA. Higher scores indicated greater subjective norms toward the perpetration of digital direct aggression, digital monitoring and control, and digital sexual coercion, respectively. Internal consistency for subjective norms was strong across all three types: digital monitoring and control (α = .89), digital direct aggression (α = .83), and digital sexual coercion (α = .84).

#### Perceived Behavioral Control

Perceived behavioral control was measured by evaluating the perceived ease or difficulty of perpetrating the 18 digital abuse behaviors within the context of a relationship (e.g., “it is easy to monitor someone’s whereabouts and activities”). Items were scored on a seven-point Likert scale ranging from 1 (*strongly disagree*) to 7 (*strongly agree*). Composite scores were computed for the three forms of DDA with higher scores indicating greater perceived behavioral control toward the perpetration of digital direct aggression, digital monitoring and control, and digital sexual coercion, respectively. High internal consistency for perceived behavioral control was demonstrated when assessed by DDA type: digital monitoring and control (α = .89); digital direct aggression (α = .96); and digital sexual coercion (α = .88).

### Data Analysis

First, descriptive statistics and correlations were conducted. Exploratory analyses revealed violations of normality across all study variables with absolute ratio values for skewness ranging from 4.61 to 30.96 and kurtosis ratios ranging from 2.04 to 81.16. The observed leptokurtic values likely reflect the low endorsement of more severe forms of DDA. As such, both Pearson’s and Spearman’s *rho* correlations were reported. To test the main hypotheses, three multiple linear regression analyses were conducted with digital monitoring and control, digital direct aggression, and digital sexual coercion as the criterion variables, and the TPB variables were entered simultaneously as predictor variables. Gender was included as a covariable (0 = man, 1 = woman). Significance was considered at *p* < .05. All analyses were conducted using SPSS v. 28.

## Results

An overview of means, standard deviations, and bivariate correlations is presented in Table 1 separated by DDA type. Emerging adults in the sample had relatively low favorable attitudes toward digital monitoring and control (*M* = 2.10, *SD* = 0.94), digital direct aggression (*M* = 1.26, *SD* = 0.45), and digital sexual coercion (*M* = 1.43, *SD* = 0.67). Similarly, there was a low perception of social acceptability and social engagement for the three types of behaviors, although perceptions were highest for digital-monitoring and control behaviors (*M* = 2.25, *SD* = 1.08), compared to digital direct aggression (*M* = 1.28, *SD* = 0.45) and digital sexual coercion (*M* = 1.41, *SD* = 0.72). In comparison, the sample reported higher perceived behavioral control for the three types of DDA (attitudes: *M* = 5.31, *SD* = 1.41; subjective norms: *M* = 5.87, *SD* = 1.53; perceived behavioral control: *M* = 5.16, *SD* = 1.67). Overall, intentions to commit digital monitoring and control (*M* = 1.59, *SD* = 0.85), digital direct aggression (*M* = 1.08, *SD* = 0.24), and digital sexual coercion (*M* = 1.18, *SD* = 0.54) in the next 4 weeks were low.

**Table 1.** Correlations for Study Variables for Digital Monitoring and Control, Digital Direct Aggression, and Digital Sexual Coercion.

Variable	*M* (*SD*)	1.	2.	3.	4.	5.
Monitoring and control						
1. Gender (women) %	85.5	—	.04	.19***	.09	.10
2. Attitudes	2.10 (0.94)	.05	—	.59***	.09	.58***
3. Subjective norms	2.25 (1.08)	.19***	.56***	—	.09	.57***
4. PBC	5.31 (1.41)	.07	.19***	.16**	—	.07
5. Intent	1.59 (0.85)	.06	.62***	.55***	.16**	—
Direct aggression						
1. Gender (women) %	85.5	—	.02	.09	.02	<.01
2. Attitudes	1.26 (0.45)	.08	—	.46***	−.01	.43***
3. Subjective norms	1.28 (0.45)	.10	.55***	—	.04	.43***
4. PBC	5.87 (1.53)	.02	.10	.08	—	.08
5. Intent	1.08 (0.24)	.03	.54***	.48***	.04	—
Sexual coercion						
1. Gender (women) %	85.5	—	.01	.13*	.05	.08
2. Attitudes	1.43 (0.67)	.02	—	.52***	.03	.51***
3. Subjective norms	1.41 (0.72)	.13*	.59***	—	.11*	.47***
4. PBC	5.16 (1.67)	.03	.10	.16**	—	.08
5. Intent	1.18 (0.54)	.05	.64***	.51***	.10	—

*Note.* Listwise *N* = 334–341. Gender: man = 0, woman = 1; PBC = perceived behavioral control. Attitudes, subjective norms, perceived behavioral control, and intent were measured on a 7-point Likert scale.

Pearson’s correlations are below the diagonal, and Spearman’s *Rho* is above the diagonal.

* *p* < .05. **p < .01. ***p < .001.

Pearson’s and Spearman’s *rho* correlations are reported in Table 1. Bivariate correlations indicated that attitudes and subjective norms were moderately associated with intentions to perpetrate all three DDA behaviors: digital monitoring and control (attitudes: *r* = .62, *p* < .001; subjective norms: *r* = .55, *p* < .001); digital direct aggression (attitudes: *r* = .54, *p* < .001; subjective norms: *r* = .48, *p* < .001); and digital sexual coercion (attitudes: *r* = .64, *p* < .001; subjective norms: *r* = .51, *p* < .001). Perceived behavioral control demonstrated a small, but significant correlation with intentions to perpetrate digital monitoring and control (*r* = .16, *p* = .002); however, when Spearman’s *rho* was calculated, this correlation was not significant (ρ = .07, *p* = .170). Perceived behavioral control was not significantly associated with intentions to perpetrate digital direct aggression (*r* = .04, *p* = .469) or digital sexual coercion (*r* = .10, *p* = .069).

Multiple linear regressions were performed to examine the relationship between the core TPB constructs and intention to perpetrate DDA. All predictors were entered simultaneously into the regression models to assess the predictive utility of each TPB construct while holding all other constructs constant; gender was entered as a control in each model (Table 2). An examination of residuals using P–P plots also indicated non-normality for all three regression models. As such, the significance of model parameters was estimated using a 95% confidence interval based on 5,000 bootstrapped samples (Efron & Tibshirani, 1986; Pek et al., 2018). All three regression models were significant: digital monitoring and control, *R*^2^ = .44, *F*(4, 333) = 65.67, *p* < .001; digital direct aggression, *R*^2^ = .34, *F*(4, 329) = 42.43, *p* < .001; and digital sexual coercion, *R*^2^ = .44, *F*(4, 336) = 65.75, *p* < .001. For digital monitoring and control, attitudes (*B* = 0.40, 95% CI [0.26, 0.53], *p* < .001) emerged as the strongest predictor of intentions followed by subjective norms (*B* = 0.23, 95% CI [0.13, 0.33], *p* < .001) such that those with more favorable attitudes and norms had higher intentions to perpetrate digital monitoring and control. Similarly, attitudes and subjective norms were significantly associated with digital direct aggression (attitudes: *B* = 0.22, 95% CI [0.11, 0.34], *p* = .002; subjective norms: *B* = 0.14, 95% CI [0.05, 0.26], *p* = .007) and digital sexual coercion (attitudes: *B* = 0.41, 95% CI [0.23, 0.56], *p* < .001; subjective norms: *B* = 0.15, 95% CI [0.02, 0.30], *p* = .041). Neither gender nor perceived behavioral control was uniquely associated with intentions to perpetrate any of the three types of DDA.

**Table 2.** Multiple Linear Regression Analyses of the Effect of Attitudes, Subjective Norms, and Perceived Behavioral Control on Intentions to Engage in Digital Dating Abuse.

Variable	*B*	95% CI for *B*		*SE B*	β
		*LL*	*UL*		
Digital monitoring and control					
Gender	−0.05	−0.23	0.13	.09	−.02
Attitudes	0.40***	0.26	0.53	.07	.45
Subjective norms	0.23***	0.13	0.33	.05	.29
PBC	0.02	−0.02	0.06	.02	.03
*R*^2^	.44				
Digital direct aggression					
Gender	−0.02	−0.06	0.02	.02	−.03
Attitudes	0.22**	0.11	0.34	.06	.41
Subjective norms	0.14**	0.05	0.25	.05	.26
PBC	0.00	−0.02	0.01	.01	−.02
*R*^2^	.34				
Digital sexual coercion					
Gender	0.02	−0.07	0.10	.04	.01
Attitudes	0.41***	0.23	0.56	.08	.52
Subjective norms	0.15*	0.02	0.30	.07	.20
PBC	0.00	−0.02	0.02	.01	.01
*R*^2^	.44				

*Note. N* = 352. Bootstrapped coefficients reported for *B*. Gender: man = 0, woman = 1; PBC = perceived behavioral control.

* *p* < .05. **p < .01. ***p < .001.

## Discussion

DDA is a growing societal problem with emerging adults commonly utilizing technology as a mechanism for perpetrating aggressions against a current or former intimate partner. The importance of factors from the TPB was partially supported by attitudes, perceived norms, and perceived control accounting for 34% to 44% of the variance in intentions to perpetrate three forms of DDA. Specifically, attitudes emerged as the strongest predictor of intentions for all three DDA types, followed by subjective norms. Perceived behavioral control failed to predict intentions for any of the DDA behaviors.

Consistent with the literature examining other cyber-based aggressions (e.g., Darvell et al., 2011; Doane et al., 2014; Pabian & Vandebosch, 2014) and the broader literature on the TPB (Rivis & Sheeran, 2003), attitudes toward perpetration emerged as the most robust predictor of intentions to perpetrate all three forms of DDA. That is, emerging adults who rated DDA behaviors as more enjoyable and more beneficial were significantly more likely to report intentions to engage in future DDA behaviors. Subjective norms also influenced intentions to perpetrate DDA, although to a lesser extent. Consistent with previous non-TPB studies examining predictors of DDA (e.g., Van Ouytsel et al., 2020) and studies on offline dating violence (e.g., Park & Kim, 2018), these findings indicate that social pressures, specifically the endorsement and actions of peers and family members, are influential on decisions to perpetrate conventional and DDA.

Contrary to expectations, results indicated no evidence that perceived behavioral control influenced intentions to perpetrate DDA. Although incongruent with the TPB, these findings are consistent with prior research examining the TPB in the context of cyber-based violence. Darvell et al. (2011) found no associations between perceived behavioral control and intentions to engage in partner monitoring using Facebook. Likewise, in applying the TPB to cyberbullying, Heirman and Walrave (2012) and Pabian and Vandebosch (2014) found no effect of perceived behavioral control on intentions or behavioral outcomes. As avid users of technologies and social media, DDA may be a behavior that most emerging adults feel they have the proficiency and opportunities to carry out (Doane et al., 2014), which is reflected in the relatively high perceived behavioral control scores observed in the current study. The present findings may provide further support to the argument that emerging adults have complete volitional control over their use of technologies and, therefore, the perceived ease with which one could commit DDA is not a deciding factor in carrying out online dating violence.

### Implications

The development of prevention and rehabilitative programs for the perpetration of DDA requires that we first identify potentially changeable factors that are related to these behaviors. The validity of these factors is further strengthened when empirical findings are consistent with a strong theoretical rationale. Based on the results of the current study, DDA prevention efforts should focus on modifying the personal and social cognitions that support emerging adults’ perpetration of DDA. Programs should increase negative evaluations of DDA by addressing the potential seriousness of these actions for both the perpetrator (e.g., legal repercussions) and the victim (e.g., adverse mental health outcomes). Furthermore, programs should address the misconception that these behaviors are normative and harmless (Stonard et al., 2017). Together, the present findings suggest that similar approaches would be effective for preventing digital monitoring and control, digital direct aggression, and digital sexual coercion.

While the present findings provide a useful foundation for understanding critical factors that contribute to different forms of DDA, the fact that perceived behavioral control, a core element of TPB, was not related to any DDA behaviors and that the models accounted for under 50% of the overall variance indicates that the TPB could potentially be enhanced by considering other theories and factors. Research in this area would likely benefit from combining factors from *general* theories like the TPB with factors that are relevant only to specific behaviors. For instance, digital sexual coercion is arguably a new form of sexual violence and exploration of sex-specific factors such as sexual deviance (e.g., arousal from coercive sex) may inform our understanding of key risk factors in this new domain. Furthermore, young adults are tech-savvy, and the current study suggests that the ease with which one can enact digital monitoring and control, digital direct aggression, and digital sexual coercion has little to no influence on young adults’ perpetration of DDA; rather, technology is the tool through which violence is enacted when risk factors, such as antisocial attitudes, are present. However, it would be advantageous to use technology-based frameworks (e.g., online disinhibition) to examine DDA perpetration and expand our understanding of how technology may promote violence via anonymity, invisibility, and other online situational factors.

### Limitations and Future Directions

The present study adds to the growing body of literature on DDA and is among the first to adopt a general behavioral theory to examine the influence of modifiable factors that may contribute to the intentions to perpetrate dating abuse through technology. However, the current study is not without limitations. First, the sample was mostly white female students from universities in Atlantic Canada. While students are a key population of interest given the high rates of dating violence on campuses (Fedina et al., 2018), the current homogeneous sample weakens our ability to generalize these findings to other populations. Future research may address this limitation by using a more balanced sample of men and women from various backgrounds, as well as the inclusion of gender-diverse individuals. In addition, most of the research into DDA has primarily focused on cis-gender and heterosexual samples or, like the present study, failed to distinguish between LGBTQ2+ and non-LGBTQ2+ members. Evidence, however, indicates that individuals in non-heterosexual relationships and non-gender conforming individuals experience elevated rates of DDA (e.g., Dank et al., 2014; Zweig et al., 2013). Accordingly, further efforts are needed to identify possible risk factors unique to these populations.

Past research has also indicated that DDA behaviors are common, with prevalence estimates as high as 93% (Leisring & Giumetti, 2014). However, emerging adults in the current study were not found to hold overly favorable attitudes toward DDA, high perceptions of social acceptability, or great intentions to perpetrate DDA. Response bias and social desirability may have played a role and influenced responses. As such, further studies may benefit from measuring and including social desirability as a covariate. Alternatively, this discrepancy may be because frequently used DDA measures, including the Digital Dating Abuse Scale (Reed et al., 2016) used in the current study, fail to distinguish between minor and severe forms of DDA which may produce composite scores that are overly influenced by those behaviors deemed more normative and appropriate. Subsequent studies should delineate between minor and severe behaviors to better assess the factors that influence particularly harmful (and potentially unlawful) DDA behaviors, such as threatening a partner or disseminating intimate images without consent.

Finally, this study utilized a cross-sectional design and did not assess the relationship between intentions and actual behavioral outcomes. Although past studies have established a general relationship between intentions and behaviors (Armitage & Conner, 2001), emerging adults typically underestimate the likelihood that they will engage in dating violence in the future given the complex and dynamic nature of intimate relationships (Kernsmith & Tolman, 2011). Therefore, a longitudinal approach is needed to elucidate the relationship between intentions and the actual, sustained perpetration of DDA behaviors within intimate relationships.

## Conclusion

Given its prevalence among emerging adults and associated harms, DDA remains an important empirical topic for exploration. The present study adds to a growing body of literature by applying a theoretical framework to DDA and suggests that related behaviors can be, at least partially, explained using the TPB. Although further investigations are needed to build upon the foundation laid in this study, prevention efforts may benefit from challenging prevailing attitudes, and social norms toward DDA. In doing so, we may be able to intervene and prevent tragic events, like that of You and Urtula, from unfolding.

## Supplemental Material

sj-docx-1-jiv-10.1177_08862605231205595 – Supplemental material for Digital Dating Abuse: An Application of the Theory of Planned Behavior Click here for additional data file. Supplemental material, sj-docx-1-jiv-10.1177_08862605231205595 for Digital Dating Abuse: An Application of the Theory of Planned Behavior by Jennifer McArthur, Julie Blais and Marguerite Ternes in Journal of Interpersonal Violence
