# Supplementary material for: Digital Dating Abuse: An Application of the Theory of Planned Behavior
Source: J Interpers Violence. 2023 Oct 21;39(5-6):1308–26. doi: 10.1177/08862605231205595 (PMC10858624; doi:10.1177/08862605231205595)
Supplement: sj-docx-1-jiv-10.1177_08862605231205595 – Supplemental material for Digital Dating Abuse: An Application of the Theory of Planned Behavior [file sj-docx-1-jiv-10.1177_08862605231205595.docx]

**Online Supplemental Materials**

**S1. Comparison of intentions between single participants and participants in a relationship.**

Aggression often continues (and may even intensify) following the dissolution of a relationship. To capture this in the current study, we required all participants to have been in at least one previous relationship and determined eligibility by providing participants with a definition of a dating partner which included a current or former partner. Intentions to perpetrate DDA were compared by relationship status (i.e., in a relationship vs. single) using independent *t*-tests (See Table 1). Analyses indicated that individuals who were single had similar intentions to engage in digital direct aggression (*M* = 1.07, *SD* = 0.22) as individuals in a relationship (*M* = 1.07, *SD* = 0.18). Likewise, individuals who were single had similar intentions to engage in digital sexual coercion (*M* = 1.15, *SD* = 0.48) as individuals in a relationship (*M* = 1.19, *SD* = 0.54). While the two groups significantly differed on intentions to engage in monitoring and control, those in the single subgroup did not necessarily default to “extremely unlikely”, having a mean of 1.43 (*SD* = .70) compared to mean of 1.68 (*SD* = .92) for those in a relationship. As such, participants who were in a relationship and participants who were single at the time of the study were retained for the remaining analyses.

**Table 1**

*Behavioural Intention by Relationship Status.*

|  | Relationship | | Single | | *t* | *p*-value | Cohen’s *d* | 95% CI |
| --- | --- | --- | --- | --- | --- | --- | --- | --- |
|  | *M* | *SD* | *M* | *SD* |  |  |  |  |
| Monitoring and control | 1.68 | 0.92 | 1.43 | 0.70 | 2.84 | .005 | 0.84 | [.08, .51] |
| Direct aggression | 1.07 | 0.22 | 1.07 | 0.18 | -0.07 | .944 | 0.20 | [-.22,.21] |
| Sexual coercion | 1.19 | 0.54 | 1.15 | 0.48 | 0.82 | .415 | 0.09 | [-.13, .31] |

**S2. Items Assessing the Components of the Theory of Planned Behaviour**

***Outcome Variable: Intentions***

Behavioural intentions were assessed by evaluating a participant’s expectation of perpetrating each of the 18 DDA behaviours using the internet or cell phone in the next four weeks on a 7-point scale from 1 (*extremely unlikely*) to 7 (*extremely likely*). Table 4 provides behavioural intention-related items on the Theory of Planned Behaviour questionnaire.

**Table 2**

*Behavioural Intention-Related Items on the Theory of Planned Behaviour Questionnaire.*

| Digital monitoring and control |
| --- |
| 1. Pressure my partner to respond quickly to calls, texts, or other messages |
| 1. Monitor my partner’s whereabouts and activities |
| 1. Send so many messages it makes my partner feel uncomfortable |
| 1. Monitor who my partner talks to and is/was friends with |
| 1. Pressure my partner for passwords to access cell phone or online accounts |
| 1. Use private information to check on my partner without permission |
| Digital direct aggression |
| 1. Share embarrassing content (photo or video) with others without my partner’s permission |
| 1. Send my partner a mean or hurtful private message |
| 1. Post a mean or hurtful public message about my partner using social media |
| 1. Spread a rumor about my partner |
| 1. Send my partner a threatening message |
| 1. Threaten to harm my partner physically |
| 1. Use cell phone or online account to pretend to be my partner |
| 1. Use information on social network sites to tease my partner |
| Digital sexual coercion |
| 1. Pressure my partner to sext |
| 1. Send a sexual or naked photo/video of myself to my partner without my partner’s consent |
| 1. Send a sexual or naked photo/video of my partner to others without my partner’s consent |
| 1. Pressured my partner to have sex or do other sexual activity |

***Attitudes***

Attitudes were measured by assessing the degree to which participants favorably evaluated the perpetration of each of the 18 DDA behaviours. Participants responded to two 7-point semantic differential scales designed to measure affective (1 - *unenjoyable* to 7 - *enjoyable*) and instrumental aspects of attitudes (1 - *harmful to me* to 7 - *beneficial to me*). Table 1 provides attitude related items on the Theory of Planned Behaviour questionnaire.

**Table 3**

*Attitude Related Items on the Theory of Planned Behaviour Questionnaire*

| Digital monitoring and control |
| --- |
| 1. To me, pressuring my partner to respond quickly to calls, texts, or other messages is… |
| 1. To me, monitoring my partner’s whereabouts and activities is… |
| 1. To me, sending my partner a lot of messages is… |
| 1. To me, monitoring who my partner talks to and is friends with is… |
| 1. To me, pressuring my partner for passwords to their cell phone and online accounts is… |
| 1. To me, using private information to heck on my partner without my partner’s permission is… |
| Digital direct aggression |
| 1. To me, sharing, embarrassing content (photo or video) of my partner with others without my partner’s permission is… |
| 1. To me, sending my partner a mean or hurtful message… |
| 1. To me, posting a mean or hurtful public message about my partner using social media is… |
| 1. To me, spreading a rumor about my partner is… |
| 1. To me, sending my partner a threatening message is… |
| 1. To me, threatening to harm my partner physically is… |
| 1. To me, using a cell phone or online account to pretend to be my partner without my partner’s permission is… |
| 1. To me, using information on social network sites to tease my partner is… |
| Digital sexual coercion |
| 1. To me, pressuring my partner to sext is… |
| 1. To me, sending a sexual or naked photo of myself to my partner without my partner’s permission is… |
| 1. To me, sending a sexual or naked photo of my partner to others without my partner’s permission is… |
| 1. To me, pressuring my partner to have sex or do other sexual activities is… |

***Subjective Norms***

Subjective norms were measured by evaluating perceptions of others’ approval to engage in DDA behaviours (i.e., injunctive norms) and perceptions of others’ behaviour (i.e., descriptive norms). Each of the 18 DDA behaviours consisted of two parallel items: one for injunctive norms and one for descriptive norms*.* All items were measured on a seven-point Likert scale ranging from 1 (*strongly disagree*) to 7 (*strongly agree*). See table 2 for subjective-related items on the Theory of Planned Behaviour questionnaire.

**Table 4**

*Subjective Norm-Related Items on the Theory of Planned Behaviour Questionnaire.*

| Digital monitoring and control |
| --- |
| 1. People who are important to me think I should pressure my partner to respond quickly to calls, texts, or other messages |
| 1. People who are important to me pressure their partners to respond quickly to calls, texts, or other messages |
| 1. People who are important to me think I should monitor my partner’s whereabouts and activities |
| 1. People who are important to me monitor their partner’s whereabouts and activities |
| 1. People who are important to me think I should send a lot of messages to my partner |
| 1. People who are important to me send a lot of messages to their partner |
| 1. People who are important to me think I should monitor who my partner talks to and is friends with |
| 1. People who are important to me monitor who their partners talk to and are friends with |
| 1. People who are important to me think I should pressure my partner for passwords to access a cell phone or online accounts |
| 1. People who are important to me pressure their partners for passwords to access a cell phone or online accounts |
| 1. People who are important to me think I should use private information to check on my partner without my partner’s permission |
| 1. People who are important to me use private information to check on their partners without their partners’ permission |
| Digital direct aggression |
| 1. People who are important to me think I should share embarrassing content (photo or video) of my partner with others without my partner’s permission |
| 1. People who are important to me share embarrassing content (photo or video) of their partners with others without their partners’ permission |
| 1. People who are important to me think I should send my partner mean or hurtful private messages |
| 1. People who are important to me send their partners mean or hurtful private messages |
| 1. People who are important to me think I should post mean or hurtful public messages about my partner using social media |
| 1. People who are important to me post mean or hurtful public messages about their partners using social media |
| 1. People who are important to me think I should spread rumors about my partner |
| 1. People who are important to me spread rumors about their partners |
| 1. People who are important to me think I should send my partner threatening messages |
| 1. People who are important to me send their partners threatening messages |
| 1. People who are important to me think I should threaten to harm my partner physically |
| 1. People who are important to me threaten to harm their partners physically |
| 1. People who are important to me think I should use a cell phone or online account to pretend to be my partner |
| 1. People who are important to me use cell phones or online accounts to pretend to be their partner |
| 1. People who are important to me think I should use information on social network sites to tease my partner |
| 1. People who are important to me use information on social network sites to tease their partners |
| Digital sexual coercion |
| 1. People who are important to me think I should pressure my partner to sext |
| 1. People who are important to me pressure their partners to sext |
| 1. People who are important to me think I should send sexual or naked photo/videos of myself to my partner without my partner’s permission |
| 1. People who are important to me send sexual or naked photo/videos of themselves to their partners without their partners’ permission |
| 1. People who are important to me think I should send sexual or naked photo/videos of my partner to others without my partner’s consent |
| 1. People who are important to me send sexual or naked photo/videos of their partners to others without their partners’ consent |
| 1. People who are important to me think I should pressure my partner to have sex or do other sexual activities |
| 1. People who are important to me pressure their partners to have sex or do other sexual activities |

***Perceived Behavioural Control***

Perceived behavioural control was measured by evaluating the perceived ease or difficulty of perpetrating the 18 digital abuse behaviours using technology. Items were scored on a seven-point Likert scale ranging from 1 (*strongly disagree*) to 7 (*strongly agree*). Table 3 provides perceived behavioural control-related items on the Theory of Planned Behaviour questionnaire.

**Table 5**

*Perceived Behavioural Control-Related Items on the Theory of Planned Behaviour Questionnaire.*

| Digital monitoring and control |
| --- |
| 1. Using the internet or a cell phone, it is easy to pressure someone to respond quickly to calls, texts, or other messages |
| 1. Using the internet or a cell phone, it is easy to monitor someone’s whereabouts and activities |
| 1. Using the internet or a cell phone, it is easy to send a lot of messages that makes someone feel uncomfortable |
| 1. Using the internet or a cell phone, it is easy to monitor who someone talks to and is/was friends with |
| 1. Using the internet or a cell phone, it is easy to pressure someone for passwords to access a cell phone or online accounts |
| 1. Using the internet or a cell phone, it is easy to use private information to check on someone without their permission |
| Digital direct aggression |
| 1. Using the internet or a cell phone, it is easy to share embarrassing content (photo or video) of someone else with others without their permission |
| 1. Using the internet or a cell phone, it is easy to send someone a mean or hurtful private message |
| 1. Using the internet or a cell phone, it is easy to post a mean or hurtful public message about someone using social media |
| 1. Using the internet or a cell phone, it is easy to spread a rumor about someone |
| 1. Using the internet or a cell phone, it is easy to send someone a threatening message |
| 1. Using the internet or a cell phone, it is easy to threaten to harm someone physically |
| 1. Using the internet or a cell phone, it is easy to use a cell phone or online account to pretend to be someone else |
| 1. Using the internet or a cell phone, it is easy to use information on social network sites to tease someone |
| Digital sexual coercion |
| 1. Using the internet or a cell phone, it is easy to pressure someone to sext |
| 1. Using the internet or a cell phone, it is easy to send a sexual or naked photo/video of myself to someone without their consent |
| 1. Using the internet or a cell phone, it is easy to send a sexual or naked photo/video of someone else to others without their consent |
| 1. Using the internet or a cell phone, it is easy to pressure someone to have sex or do other sexual activities |
